# Sustainable tourism policies in Peru and their link with renewable energy: analysis in the main museums of the Moche route

**DOI:** 10.1016/j.heliyon.2021.e08188

**Published:** 2021-10-20

**Authors:** F. Calderón-Vargas, D. Asmat-Campos, P. Chávez-Arroyo

**Affiliations:** a Universidad de Almería, Departamento de Economía y Empresa, Almería, Spain; b Universidad Privada del Norte, Dirección de Investigación e Innovación, Trujillo, Peru; c Universidad Nacional de Trujillo, Facultad de Ciencias Sociales, Trujillo, Peru

**Keywords:** Sustainability, Renewable energies, Solar energy, Wind energy, Sustainable museums, Sustainable tourism

## Abstract

Tourism activity in Peru has been experiencing significant growth in the last ten years, positioning this economic sector as the third largest contributor to the National Gross Domestic Product (GDP). Likewise, Peru has a high ecological and climate diversity, which makes it the possessor of renewable energy potential, specifically solar and wind power. The rapid growth of tourism is leading to generating prospects for becoming a sustainable destination. In this sense, it is important to understand and evaluate the Peruvian legislative framework for sustainable tourism and the current state of the implementation of the scenarios provided by the governmental entity in terms of sustainability, and its link with tourism activity. Based on what has been described, this study is aimed at evaluating the four most relevant museums in the northern part of Peru; in addition, it contributes to the studies that exist at the intersection of tourism and sustainability in the chains of activities related to tourism and calls for rationality applied to tourism management in this region of Latin America. The results of the literature review of the Peruvian legal framework reveal a lack of specific laws and regulations on sustainable tourism; on the contrary, there are policies in force that contribute to promoting the development of sustainable tourism. The quantified evaluation of the solar and wind potentials of the geographical area under study indicates the minimum renewable energy potential necessary for its transformation and use in the development of sustainable museums and its contribution to sustainable tourism.

## 1 Introduction

Tourism activity in the last decade is having a strong impact on the international economy as it represents 10% of gross domestic product (GDP) and 10% of global employment (OMT-a, 2019). Tourism is also known to generate growth, development, to modernize remote areas and to accelerate cultural development (Tian et al., 2021). Only in 2019, approximately 1.5 billion international tourist trips were recorded, an increase of 4% more than the previous year (Michalena et al., 2009). Consequently, this growth has positive effects but also negative effects that threaten heritage, cultural identity, and well-being (Grilli et al., 2021). In the environmental field it generates more emissions of greenhouse gases, increasing the carbon footprint (Sun and Drakeman, 2020). A study carried out by WTO and UN reveals that tourism contributes 5% of all CO_2_ produced by man (OMT-a, 2019), and between 50 and 60% of carbon emissions are indirectly related to this industry (Dwyer et al., 2010) with a strong growth rate (3% per year), hoping that tourism emissions will increasingly contribute to the carbon budget of global economy between 2020 and 2060 (Sun and Higham, 2021). This has become a concern, due to its impact on environmental pollution and the higher level of demand for fossil fuels and energy intensity (Katircioglu, 2014). In 2009, an ambitious tourism reduction target was proposed, with the aim of reducing emissions of this sector from 25% to 30% by 2020 and to 50% by 2035 from the base year 2005. Despite this ambitious vision, very few countries have identified cohesive mitigation strategies related to tourism and even fewer have implemented such policies (Sun and Higham, 2021). That is why the need for responsible growth of the sector is defended through sustainable tourism development, since the growing environmental pressure urges the world to create strategies to cushion this problem from different sectors.

It is necessary to bear in mind that, after the crisis generated by the COVID-19 pandemic, we have to project and restructure our form of tourism management in the different destinations, directing and reorienting it under the parameters of sustainability (Bertella, 2020), to balance tourism income (Sustainable Development Goal N° 8 ″Decent Work and Economic Growth") and its impact on climate change (Sustainable Development Goal N° 13 ″Climate Action") (Sun and Higham, 2021). Today, tourism has a central place in global development policies, and can still achieve greater political recognition and have an effective impact on the 2030 Agenda and its 17 Sustainable Development Goals (OMT-b, 2019). The World Tourism and Travel Council set targets to reduce carbon emissions by 25–30% by 2020 and 50% by 2035 (Tian et al., 2021). Likewise, the Paris Climate Agreement aims to stabilize global average temperatures below +2 °C in relation to pre-industrial levels on the basis of the commitment of 196 countries (Sun and Higham, 2021). In the light of these prospects, it is important that each country be politically prepared to improve governance and to address the negative effects the sector may cause, that governments take measures to reorient the development of the activity by implementing policies that support the promotion of sustainable tourism activity, since this type of tourism takes into account current and future repercussions, from economic, social and environmental perspectives, to meet the needs of visitors, industry, environment, and host communities (OMT, 2005). Consequently, each country, as a strategy, must strengthen the implementation of policies and general guidelines that guide the actions of the State in the long term in order to achieve the well-being of the people (CEPLAN, n.d.), since (Becken et al., 2020) based on the extensive review of 101 documents of policies from 61 countries, they point out that tourism climate policy is largely ignored within governance and climate change policy processes. One way to make tourism increasingly sustainable, in addition to working together with the community to improve its quality of life, is to have environmental parameters that contribute to reduce energy consumption, and for this our best ally is the use of renewable energies, energies that we find in nature in unlimited quantity, and whose impact on the environment is practically zero or reversible (Enel, 2018). Renewable energy has shown to have clearly fewer local, regional and global environmental impacts than conventional energy sources (PNUMA, 2003). In addition, tourism is one of the driving forces for both economic growth and environmental sustainability, so the interaction between pollution and renewable energy consumption requires more attention (Sarpong et al., 2020). On the other hand, an added value is given to destinations because tourists are willing to pay for activities that are likely to promote environmental quality (UNEP, 2011). According to UN, more and more travelers want to do tourism without harming the planet. Today's tourists want to collaborate with the countries and communities they visit (ONU, 2017).

Various authors support the importance of involving and directing tourism to the use of renewable energies. Various studies consolidate the nexus between tourism development and environmental quality, linking the role of renewable energy consumption and income (Calderón-Vargas et al., 2019; Tian et al., 2021). There are also studies on the application of renewable energy technologies in the development of low-carbon rural tourism (Chen, 2011). Others investigate the roles of international tourism and renewable energy in the environment, focused on evidence from Asian countries (Zhang and Liu, 2019); there are also documents analyzing the relationship between tourism as a tool for nature conservation and the conflict between renewable energy projects and nature protection in Iceland (Ingó lfsd ó ttir and Gunnarsd ó ttir, 2020) and the development of sustainable tourism, by using a multi-criteria analysis on renewable energies in the Mediterranean islands (Michalena et al., 2009).

However, within the research topics there are more specific studies, linked to the hotel category, since, together with transport, they are the two activities with the greatest demand for energy in tourism sector. Transport accounts for 94% of energy use, followed by accommodation with 3.5% (Calderón-Vargas et al., 2019). Therefore (Dhirasasna and Sahin, 2020) applies a system dynamics model for the adoption of renewable energy technologies in hotel sector. (Navratil et al., 2019) addresses visitors' preferences about renewable energy options in "green" hotels. However (Michalski et al., 2019), focuses the study on the role of the supply of renewable fuels in transport sector in a future decarbonized energy system, and (Teske et al., 2018), in scenarios of high penetration of renewable energies and their implications for urban energy and transport systems.

This study is proposed because most studies of renewable energies are focused on researching on hotel and transport sectors, but they have neglected to focus on other sectors that, despite their not massive existence, have a high energy consumption. In this specific case, reference is made to museums, which due to the air conditioning of the large exhibition halls, the general space and the high rate of air change due to the influx of visitors, museums are usually large energy consumers due to the need to provide strict moisture control, and, in addition, they often have specific and/or different heating and cooling requirements for their artifacts and collections (Cadelano et al., 2019). Most of the museums, despite being subject to architectural protection or strict requirements of interior microclimate, must comply with the current norms on energy efficiency, thermal comfort and safety (Cadelano et al., 2019). The more economic activities join the cause of contributing to the sustainability of the country, the closer it is to achieve the goal of being a member of the Organization for Economic Cooperation and Development (OECD) in ten years. Thus, it is forced to carry out a series of reforms and improvement of the institutions, that allow to gain competitiveness, transparency and propel to the best international standards (Gamio, 2016). In this regard, Peru should gradually move towards a "cleaner" growth, which would generate fewer emissions and would not compromise economic and social development, improving its competitiveness and productivity. However, we must do it on a technical basis and gradually using clean technologies, starting from those offering us the lowest costs (Gamio, 2016). We have to take advantage of the country's exceptional wind resources and great solar energy potential, thanks to its geographical, climatic (MINEM, 2001) and geothermal characteristics for the construction of renewable power plants throughout the country (MOCICC, 2020), because, according to a study carried out by IRENA, in coordination with the Ministry of Energy and Mines of Peru, in 2014 the study "PERU: Evaluation of the State of Preparedness for Renewable Energies" was published, which evaluates the situation and evolution of renewable energies in Peru until 2014. In that report, the Peruvian State reported that the renewable wind potential feasible to be converted into electricity was equal to 22,500 MW. The Wind Atlas of Peru estimated that in Peru there are 20,493 MW of exploitable wind resources out of a total wind resource of 28,395 MW, which is interesting for the installation of wind power generation systems (MOCICC – Movimiento Ciudadano frente al Cambio Climá tico, 2020).

The objective of this research is to understand and evaluate the Peruvian legislative framework for sustainable tourism and link it with policies on the use of renewable energies; for this, a sustainability analysis is also being developed based on a quantified evaluation of the solar and wind potential of the geographic sector under study, this as an alternative potential for its application in the development of sustainable museums and its contribution to sustainable tourism. The study is aimed at the four most relevant museums in the northern region of Peru.

## 2 Methodology

### 2.1 Research context

Peru is a country with growing tourism development, the presence of one of the wonders of the world (Machupicchu) has been attracting a large influx of foreign tourists, who in turn tend to complement their experience visiting the northern part of Peru, a place where the so-called "Moche Route" is located, which was formerly the headquarters of the Moche Culture (150–700 d.C), within its tourist attractions, this area is home to four important museums in the country, being an excellent scenario to explore renewable energy potential and motivate its use by adapting to sustainable museums. Despite being an ideal and preferred destination for the commercialization of tourism services, the tourism industry in the areas in question faces sustainability challenges and the lack of governmental strategies in terms of sustainability.

### 2.2 Information gathering and analysis

Data from scientific articles indexed in prestigious databases such as Scopus and Web of Science have been used to carry out this manuscript; as well as national and supranational official organizations: the World Tourism Organization (UNWTO), Ministry of Foreign Trade and Tourism of Peru (MINCETUR), Ministry of Energy and Mines of Peru (MINEM) and Peruvian state laws; which has allowed us to have reliable and verifiable information, to know the Peruvian legislation in terms of sustainability and renewable energy, tourism trends, as well as the importance of sustainable tourism at an international level. The use of data from various sub-organizations of the United Nations has also been used, which helped us to reinforce and sustain our proposal and study in the environmental field; on the other hand, MINCETUR figures have been used to obtain tourism data in figures, deciphering the main museums of the Moche Route, the influx received by each museum studied in the last ten years, and the profile of the tourist who visits the Moche Route. Regarding the study of renewable energy potential, our analysis was based on data available from the National Aeronautics and Space Administration (NASA) and the Geographic Information System (GIS) provided by the World Bank's SOLARGIS and simulation software energy provided by EnAir.

It is worth mentioning that due to the situation caused by the COVID-19 pandemic, there are no updated figures that reflect tourist activity in 2020, this despite the fact that internal tourism activities have been gradually resuming from October to December of the same anus; however, due to the arrival of the second wave of the pandemic in Peru (January 2021), this activity has been restricted again in a large percentage, which is why the figures presented in the investigation are limited to 2019, to present a correct behavior in the annual statistics.

As described, this research was carried out through a systematic review of the literature. This method is systematic because the literature is reviewed and articles are selected for inclusion, in a way that is reproducible (Hazemba and Halog, 2021).

Building the investigation follows five key steps. The first is to carry out a sufficient bibliographic review on three key concepts of the subject: sustainable development policies, sustainable tourism and museums. The systematic review aims to adjust the scope of the study, and identify the points highlighted in previous works. The second step is to carry out a systematic synthesis of the Peruvian legislative framework on sustainable tourism and the policies on the use of renewable energies, for this the information platforms of the virtual portal of the Government of Peru were accessed, where the laws, decrees are published and standards. This approach is important since the analysis revolved around the legal characteristics of the country under study. The third step consists of using the information presented in the literature review as a systematic mapping of government policies within the legislative framework. The fourth step is linked to a quantitative research, this because data has been collected using high precision programs to evaluate the renewable energy potential of the sectors under study. The fifth step is dedicated to the discussion of the results of the systematic review, and based on what was obtained in the fourth step, to publicize the relevance of the topic so that it can be used.

Regarding the government policy framework, governments often develop national development plans, strategies and legislative frameworks to meet the perceived needs and priorities of their citizens (Hazemba and Halog, 2021).

These plans also influence the areas of support provided by development partners. They are often a combination of sectoral strategies, plans and other cross-cutting development issues, and typically focus on economic growth and poverty reduction (Hazemba and Halog 2021); (ONU, n.d.-a).

Every step is an opportunity to incorporate environmental management objectives (ONU, n.d.-b) and therefore requires a sustained programmatic approach that is tailored to the circumstances of a country (PNUMA, 2009).

## 3 Tourism and its legislation related to sustainability in Peru

The motivation for this study comes from the fact that at a global level the effects of climate change, the damage caused and the impact on the population and nature are already beginning to be shown, topics of high interest for international entities that have been providing year after year statistics and projections on the effects, which causes great concern, that is how various governments have been promoting mitigation policies, and now in addition to adaptation policies, however, in underdeveloped countries, these issues are still superficially addressed. Given this, the need arises to investigate government policies in Peru, a country in Latin America, with a high potential for renewable resources, in addition to a growing tourism context, and evaluate if there is any contribution at the level in this line of development. mitigation against this threat to the planet (Benites-Lazaro and Andrade, 2019; Cohen et al., 2021). It should be noted that tourism is currently being focused on a sustainable tourism context, thus adding to the contribution. Various countries have already been contributing in the practice of sustainable tourism (De Graaf et al., 2014; Sharif-Askari and Abu-Hijleh, 2018; Vaseghi et al., 2020), considering a start-up of new systems based on the use of renewable energies (Chandra & Kumar, 2021; Lee et al., 2021; Quevedo et al., 2021), promoting policies that link both criteria (Byrne et al., 2007; Dwyer et al., 2016; Streimikiene et al., 2012; Wanner et al., 2020), however, in the context under study, a question arises: Peru, despite having renewable energy potential, ¿why does it not develop and/or promote it? The answer lies in evaluating the context in which this research presents.

Tourism growth of Peru in the last ten years is remarkable. According to data from the UNWTO, international tourism fell by 5% in Latin America in 2019 (OMT-b, 2019). By contrast, Peru in 2019 received 4,371,787 tourists, meaning a growth of 4% and an approximate foreign currency income of USD 5,300 million coming from receptive tourism (DIARIO GESTIÓ N, 2019). Likewise, in terms of national tourism according to MINCETUR, in 2019 there have been 48.6 million national trips for tourism (a tourist can make more than one trip in the year). These results are only covering Peruvians who travel for leisure purposes (MINCETUR, 2019). For this, tourism has become the third economic activity and the generator of almost 4% of the national GDP (Marsano, 2019).

Peru has a diverse tourist offer which makes it a country with good potential to continue growing tourism, then it is essential to strengthen an orderly and strategic development in order to move towards the pillars of tourism sustainability, contributing from this sector to meet the objectives of the 2030 agenda, where Peru has been one of the leading countries in the elaboration of that agenda, and also led the second round of consultations to define the seventeen Sustainable Development Goals (ONU, n.d.-b). For this, it is essential to have good legislation that is the basis for the management of tourist attractions, resources, and destinations by governmental and private agencies, in order to safeguard their historical, endogenous cultural, and natural legacies, as well as to involve local population in activities with the purpose of improving their quality of life, this is stated by (Tyllianakis et al., 2019), where the implementation of policies that promote sustainable tourism, contributes to the creation of new jobs and promotes local culture, projecting great growth for 2030.

It details the main policies implemented by the Peruvian state, which contribute to strengthen sustainable tourism development, having as reference the main law that governs the activity in the country. It is worth to mention **Law N° 29408, General Tourism Law**, which contains the legal framework for the development and regulation of tourism activity; in turn, it aims to promote, encourage and regulate sustainable development of tourism activity at national, regional, and local levels. It defines priority tourism development areas, establishing their necessary sustainable management between the public and private sectors. It promotes mixed financing schemes that consider public and private investment in order to contribute to the improvement of the quality of life of the host population and the transformation of tourism resources into sustainable tourism products in accordance with the guidelines of the National Strategic Tourism Plan - PENTUR (MINCETUR, 2009). With the contribution of both the public and private sectors in PENTUR, they set 9 strategic objectives for 2021, with the aim of improving the sector's competitiveness. These strategic objectives include strengthening sustainable tourism management processes (8), as well as promoting institutional strengthening of State entities and destination management institutions (9), in order to position Peru as a destination at national and international level and the tourist activity as one of the main economic activities of the country (3). In PENTUR 2025, priority is given to forging actions to move towards sustainable tourism, proposing that Peru develop a tourism based on a multi-thematic, sustainable (Tasso et al., 2015).

The **Organic Law of Municipalities N° 27972** regulation that shows the projection of linking sustainability with tourism activity. In this law, local governments are empowered to promote sustainable local tourism; also decrees the promotion of sustainable tourism, regulating the services intended for this activity, in cooperation with the competent entities. It involves participation and coordination between public entities and the private sector in order to adopt effective measures to prevent, control and mitigate the deterioration of the environment and its components, in particular, the natural resources and assets of the Nation's Cultural Heritage, as a result of the development of infrastructure and tourism and recreational activities, which are likely to have negative impacts on them (Perú, 2003). **Law N° 27790, Law on Organization and Functions of the Ministry of Foreign Trade and Tourism**, which aims to promote and regulate tourism in order to promote its sustainable development. It promotes the rational and sustainable use for tourism purposes of the Nation's Cultural and Natural Heritage (MINCETUR, 2022). The **Environmental Policy of Tourism Sector (Ministerial Resolution N° 195-2006-MINCETUR-DM)**, defines the purposes, principles and provides the guidelines of the environmental policy in tourism sector. It establishes the framework of reference for the definition and achievement of environmental goals and targets while respecting the needs of current populations. It promotes the proper use of tourism resources, the implementation of measures for the prevention of environmental pollution and the conservation of biodiversity (MINCETUR, 2006). **Law N° 28611, General Environmental Law,** has also been taken into account, since it establishes effective measures to prevent, control and mitigate the deterioration of the environment, natural resources and assets of the Nation's Cultural Heritage, as a result of the development of infrastructure and tourist and recreational activities, which may have a negative impact on them. It identifies marine and coastal areas as spaces for the development of sports, recreational and ecotourism activities (MINAM, 2005). Likewise, the **Regulation of the Registry of entities authorized to prepare environmental impact studies and other environmental management instruments applicable to the activities of Tourism Sector (Supreme Decree N° 010-2008-MINCETUR)** establishes the rules for the registration and renewal of the registry of authorized entities, being national or foreign ones, to prepare environmental impact studies and other environmental management instruments, applicable to the activities of Tourism Sector (MINCETUR, 2008). **Law N° 29763, Forestry and Wildlife Law**, establishes concessions for the development of ecotourism, seeking to generate low impact on the natural environment, and allowing for beneficial socio-economic participation for local populations (MINAM, 2011). Finally, **Law N° 26834, Law of Protected Natural Areas**, which aims to forge tourist awareness and to incorporate in the instances the conceptualization of sustainable tourism and environmental management motivating respect and enhancement of socio-cultural identity. It promotes the development of sustainable tourism companies. It conducts studies to estimate tourism impacts on the environment (MINAM, 1997).

In this sense, the research carried out allows us to infer that Peru is beginning to project itself in the creation of policies that include sustainability, but there is still a need to create a specific law for sustainable tourism, a law that serves as a guide to both the public and private sectors to direct actions towards sustainable development. In Latin America there are countries that have already launched this initiative, such is the case of Colombia that in 2020 has created its Sustainable Tourism policies, with the aim of articulating all the actors in the sector around the objective of developing a sustainable tourism, since it is a potential opportunity to transform itself, consolidating itself as an economically profitable and viable business, but also as a vehicle for social development, a means of protecting the communities way of life, and an instrument for the conservation of the environment, biodiversity, ecosystems and natural resources of the country (Minicomercio, 2020).

On the other hand, it should be taken into account that the World Tourism Organization regularly prepares reports for the United Nations General Assembly with updated information on sustainable tourism policies of UNWTO Member States and Member States of the United Nations, as well as the competent agencies and programs of the United Nations system (OMT, 2020).

## 4 Renewable energy and its contribution to sustainable development

One of the priorities of the 2030 agenda is to reduce gas emissions that are responsible for the increase of the greenhouse effect. As a key strategy, it has been proposed within the Sustainable Development Goals (Sustainable Development Goal 7: Affordable and Clean Energy), to ensure access to affordable, safe, sustainable, modern and clean energy for all, with a view to enhance international cooperation by facilitating access to research and technology on clean energy, including renewable sources, energy efficiency, advanced and cleaner technologies, also to promote investment in energy infrastructure and clean technologies. It also provides the motivation to expand infrastructure and to improve technology to deliver modern and sustainable energy services in all developing countries, in particular the least developed countries (ONU, n.d.-b).

As Peru is one of the leading countries in the elaboration of the 2030 agenda, as well as one of the most active partner countries and collaborator of the OECD, where five key areas were created, including economic growth and care for the environment, it has to be legislatively prepared to tackle, from the highest bodies to local governments, the implementation of services to cover energy demand based on the use of renewable energy, as well as to motivate the private sector to contribute to this cause, since the main sources of energy used today are polluting and are being rapidly depleted. We must take into account that Peru, as a country, has an important energy potential which must be efficiently used since it only uses 2% of the total existing wind resources, despite of having exceptional wind, solar and geothermal resources for the construction of renewable power plants throughout the country, especially in the regions of Cajamarca, Lambayeque, Piura, La Libertad and Ancash, in the north of Lima, as well as in the department of Ica and the provinces of Caravelí and Camaná in Arequipa Region (MOCICC, 2020). On the other hand, Peru is one of the countries with the largest solar resource in the world due to its proximity to the equatorial line, having many more hours of sun during most of the year, which favors the construction of solar thermoelectric generating systems. The most important technical and economic condition for the installation of thermoelectric solar systems is to have annual direct solar radiation, not less than 2000 kWh/m^2^, and southern Peru has annual direct solar radiation values higher than 3000 kWh, being Peru's total geothermal potential 2860 MW (MOCICC, 2020).

Renewable energy is considered one of the most promising ways to support sustainable development (Shen et al., 2020), it is because of that strategically Peru is directing its policies to deal with the problem of electricity shortages in rural areas, vulnerable communities, remote areas, etc. Through the implementation of renewable energy, which is supported by the **General Rural Electrification Law N° 28749**, which aims to establish the regulatory framework for the promotion and the efficient, sustainable development of electrification in rural areas, isolated localities, and border areas of the country. This law gives priority to the development of rural electrification projects, which must give priority to the exploitation and development of renewable energy resources from solar, wind, geothermal, hydraulic and biomass sources in the national territory, as well as to their use for sustainable development in rural areas (Osinergmin, 2015). On the basis of this law, the National Rural Electrification Plan is in force until 2025 and its fundamental role is to promote that more families in rural areas have both conventional electricity and that one based on the use of renewable energy. The Peruvian government, through its ministries of Energy and Mines, Environment, and, in turn, the Legislative Branch, have highlighted the importance of renewable energy in Peru. It has also celebrated agreements with international entities, such as the Inter-American Institute for Cooperation on Agriculture - IICA, and ACCIONA Microenergy Peru - AMP, whose objective is to facilitate the access to renewable, thermal and electrical energy technologies. In addition, there is cooperation between public universities and the state. Thus, the National Engineering University, through its Center for Renewable Energies (CER-UNI), has been implementing photovoltaic systems in the tourist area of the communities of the Uros, Taquile, Amantaní, in Puno Region (Calderón-Vargas et al., 2019).

In order to involve more economic and social sectors in the use of renewable energy, it would be essential to create legislation that encourages the implementation of renewable energy in each socio-economic area of the country. As a sustainable alternative to the activity, it would be necessary a policy that encourages the construction and implementation of new infrastructures considering sustainable elements, such as the use of clean energies that are obtained from natural sources, practically inexhaustible, causing minimal impacts on the environment during its transformation. Currently, few tourist service providers or attractions opt for the implementation and use of clean energy, often using conventional sources (coal, water, hydrocarbons, nuclear fuels), not taking into account the great natural potential that they have, which would contribute to reach the objective of sustainability. At the moment, there is only the **National Energy Policy of Peru 2010–2040 Supreme Decree Nº 064-2010-EM**, which is general and can indirectly involve other activities and economic sectors in the country. This policy has nine objectives that impel the country to achieve the implementation of an energy system that meets national demand and promotes sustainable development. Objective six (6) specifically promotes the development of the energy sector with the aim of generating a minimum environmental impact with low carbon emissions in a framework of Sustainable Development, encouraging development, use of clean energy and low-emission pollutant technologies to prevent the biodegradation of resources. It also seeks to establish measures for the mitigation of emissions coming from energy activities, promoting energy projects that help to obtain benefits based on emission reduction certificates (CERs), to promote the intensive and efficient use of conventional and non-conventional renewable energy sources, as well as the distributed generation (MINEM, 2010). Peru also focuses on creating strategies to strengthen **The Intended Nationally Determined Contribution** (INDC), where the Peruvian iNDC considers a 30% reduction in Greenhouse Gas (GHG) emissions projected for 2030, as part of a Business as Usual (BaU) scenario. The Peruvian State considers that a 20% reduction will be implemented through investments and expenditures with domestic, public, and private resources (unconditioned proposal), and that the remaining 10% will be subject to the availability of international external financing (MINAM, 2015).

In 2017, to strengthen the purpose, the Peruvian Renewable Energy Society was created, a non-profit civil association that brings together companies and organizations that are committed to the development of non-conventional renewable energies. Their mission is to encourage the development of electric power generation in Peru from renewable resources. Its vision is to be the organization that helps Peru to generate renewable energy, in a decentralized way based on a diversified matrix.

In this area, the literature reviewed concludes that Peru has embarked on implementing renewable energy strategies emphasizing rural electrification, but not, it is firmly proposing strategies aimed at diversifying its implementation in economic areas, allowing it to have more relevance and even growth. economic as stated (Qudrat-Ullah and Nevo, 2021), who asserts that the adoption and development of renewable energy will lead to increased economic growth in Africa in both the long and the short term; likewise (Ullah et al., 2021) in his study of 122 countries, he shows that there is a significant and positive relationship between renewable energy and economic growth in the high regime. On the other hand, we can rely on examples of success in the implementation of renewable energies, the development agenda prescribes the application of renewable energy policies as a pillar of energy security and sustainable development in both developed and developing countries (Gatto and Drago, 2021) Brazil being a model country in Latin America since they perform better in the consumption of renewable energy; likewise, it is confirmed that green countries, for example, the Nordic region, maintain an attitude of high consumption of renewable energy (Gatto and Drago, 2021).

## 5 Linking renewable energy and tourism in Peru

As previously mentioned, Peru is a country that has great tourist potential thanks to its landscapes, culture and geomorphological characteristics that have allowed it to have 84 of the 117 life zones in the world, for which there is a greater responsibility for the care and preservation of these areas, it must be taken into account that the most moderate scenario of climate change can be seriously damaging; Likewise, the country's potential growth would be negatively affected, since several activities with great economic potential depend on the ecosystem resources that this diversity provides (such as the hydroelectric sector, agriculture, livestock and tourism); likewise taking into account the Sustainable Development Goals, goal 13 refers to take urgent measures to mitigate climate change and its effects. In this case, tourism activity actively contributes to climate change, both negatively and positively; on the negative side we have that between 50 and 60% of carbon emissions are indirectly related to this industry (Dwyer et al., 2010), and that according to the study by (Becken et al., 2020), after analyzing the policies of 61 countries, the climate policy of tourism is largely ignored within governance; The positive aspect is that there is increasing impetus to the development of sustainable tourism which advocates the protection of the environment, natural resources and generates benefits for the local community (Tyllianakis et al., 2019). Given the above, it should be interesting that sustainable tourism has a leading role in response and contribution global response to climate change by reducing conventional energy consumption and opting for the use of renewable sources in both rural and urban areas, since 76% of the population lives in urban areas, with an annual growth rate of 2.1%, while rural areas have grown at a rate of 0.01% per year (MINAM, 2015). Then, it is essential to consider the vulnerability of cities and to promote the concept of "resilient cities" as management units associated with climate risk. In view of this, in the last decade, Peru has signed international commitments, such as the Paris Agreement, in 2015 (ratified in 2016), through which it is committed to develop strategies for both mitigation and reduction of emissions, and adaptation to climate change. The Peruvian Expected and Determined Contribution at National Level, presented to the United Nations Framework Convention on Climate Change (UNFCCC), contemplates a 30% reduction in the projected greenhouse gas emissions for 2030, as a part of a *Business as Usual* (BaU) scenario, starting in 2010. From this reduction proposal, 12% belongs to the energy sector (MINAM, 2015). It should be noted that, according to the Tourism Observatory of Peru (OTP), tourism has become the third most important economic activity in the country (OTP, 2019), generating a significant income, both for international and national tourism, emitting and receptive, so the more the sector grows, the more likely it is to generate a negative impact on environmental sustainability, since it causes more consumption of services that are essential for meeting visitors' needs. For this, taking into account the principle of sustainability, tourism policies in every country should aim to combat the consequences of climate change caused by "chimney-free industry", such as the disappearance of beaches due to sea level rise, among others. A feasible alternative is the use of renewable energy to mitigate the consequences and that brings, as a benefit, the saving and efficient use of energy. The result would be a competitive tourist offer through the responsible use of resources.

On the other hand, they should contribute to the country becoming the most competitive destination, since, according to the tourist profile of MINCETUR, the demands of visitors have changed. More and more visitors are interested in environmental preservation and sustainability, being attracted by nature-type activities. This is a result of the awareness of the care of the planet and other environmental factors.

In this regard, it is necessary to refine a new concept, involving the participation of renewable energies and tourism, since more and more tourists demand ecological and sustainable places. Besides, the vast majority of tourist attractions are located in areas with a large number of natural resources and, in particular, with favorable climatic and environmental conditions for the use of renewable energy sources, such as solar, wind, hydraulic, biomass, tidal, among others; and, from them, to cover energy requirements in a sustainable way.

For this reason, several previous studies indicate that the implementation of renewable energy technology can be applied in the development of tourism, contributing to the reduction of carbon (Chen, 2011); likewise (Waheed et al., 2020) it is committed to the long-term cointegration between tourism, renewable energy, capital and economic growth in the case of Saudi Arabia, since tourism and renewable energy are very crucial for economic growth in this place. (Khan et al., 2019) recommends linking financial development (where tourism is immersed), with renewable energy and ecological technologies, increasing renewable energy in America. With this strategy, we as a country would be heading towards the creation of new tourism products generating more attractiveness in visitors, boosting the economy by reducing carbon emissions.

## 6 Importance of the Moche route

Tourism industry has great importance in the productive strategy of any country, because it contributes with its economic growth, seeks to generate income and employment, promotes the development of infrastructures, stimulates profitable national industries (accommodation establishments, food service, transport system, handicrafts), etc.

According to the 2019 Tourism and Travel Competitiveness Report, Peru had just over 4 million international tourists, ranking 49^th^ worldwide, improving two positions over the previous year, with natural (position 13) and cultural (position 27) resources as its strengths (WEF, 2019). Peru seeks to move up positions in the aforementioned ranking of world tourism by complying with the indicators that allow the sustainable development of travel and tourism sector, observing an evolution in the last four years. In fact, the country has been improving positions and scores since 2015 (when it ranked 58^th^) (El Comercio, 2019).

Peru hosts important natural and cultural attractions, but Machu Picchu exerts great power of attraction for national and international tourism, more than any other destination in the country (Figueroa, 2018). According to the Travel and Tourism Competitiveness Report, the country has done a good job of promoting and preserving its cultural heritage, which has resulted in an increasing number of visitors to its world-famous sites. However, it may have been a victim of its own success, focusing exclusively on a few points of interest, rather than extending development initiatives to wider areas (OMT, 2016).

Consequently, as a productive strategy, in recent decades, the Peruvian Government has sought to diversify tourist offer by enhancing tourist attractions of other regions in the country, such as the North Coast of Peru, in order to present a diverse and renewed country to attract the international tourist gaze towards other destinations (Gonzales, 2017).

In this context of trying to diversify the tourist offer, the Moche Route was created in 2008. This is a tourist destination that includes the coastal strip of the regions of La Libertad and Lambayeque and that were territory of one of the most important ancestral cultures of this continent, the Moche or Mochica culture (DIARIO GESTIÓN, 2019). According to the information collected by the National Center for Strategic Planning (Ceplan), the Moche Route receives about 100,000 foreign visitors and 500,000 Peruvian visitors per year, generating US$140 million of revenue (El Comercio, 2019).

Its objective is to promote research, conservation and public use of cultural and archaeological heritage on the north coast of Peru (Uceda et al., 2017). The Main Sales Proposal (MSP) of the Moche Route destination is a "Cultural – Archaeological Tourism", and is mainly formed by the following attractions: Bruning Museum, Royal Tombs of Sipan Museum, Archaeological Complex of Sipan, Huaca Rajada-Sipan Museum, El Brujo Archaeological Complex, Cao Museum, Chan Chan Citadel, Huacas del Sol y la Luna, Huaca Arco Iris, Sican Archaeological Complex, Túcume or Valle de las Pirámides The tourist destination complements its main attractions with other cultural and natural resources (Aguilar, 2011).

The museums of the Moche Route that recorded the greatest influx according to MINCETUR are: Royal Tombs of Sipan Museum, which exhibits gold objects of the funeral goods of the Lord of Sipán, and in 2019 it received 185,388 visitors. The second busiest place in 2019 was the On-site Museum Huacas de Moche, which received 136,653 visitors. In third place, the Túcume Site Museum, which shelters a thousand ceramic, metal and textile objects, and, according to MINCETUR, in 2019, it received 70,471 visitors. Fourth, the Cao Museum, which exhibits the mummified body of a Moche dignitary, and, according to MINCETUR, in 2019, it registered 66,040 visitors (MINCETUR, 2020) (MINCETUR, 2022).

## 7 Renewable energy potential in the main museums of the Moche route

### 7.1 Renewable energy potential

This section considers the assessment of solar and wind potential in the geographical locations where the museums under study are located. This as an alternative source of energy which can be added to the demand for a museum, with the aim of reducing conventional energy consumption, consolidating a new concept of sustainable museum, and reducing greenhouse gas emissions, including CO_2_.

The information has been acquired from high-precision sources, such as the National Aeronautics and Space Administration (NASA), the Geographic Information System (GIS) provided by SOLARGIS of the World Bank and the simulation software - Wind energy assessment provided by EnAir.

#### 7.1.1 Solar potential

Solar energy is considered one of the sources of life for various living beings that inhabit the Earth. Currently, there are technologies that have managed to be imperative with the objective of transforming this type of renewable energy into electrical and thermal energy, with applications ranging from home systems (Langer and Volling, 2020; Lu et al., 2020; Munzke et al., 2021) to industrial systems (Joshi et al., 2020) (Xu et al., 2019). This is how, currently, the use of alternative energies (Comakli et al., 2008; Patchell and Hayter, 2021), is being implemented with greater emphasis. Europe is one of the most successful continents in supplying electricity through the use of photovoltaic panels (Frank et al., 2021); solar architecture is also appearing in innovative designs that aim to establish a more efficient system of lighting and heating.

The geographic location is important to determine the solar potential of the place; this, by means of a series of measurements that will allow to make calculations in order to determine the degree of dependence of this type of source, and the diverse options that its use imply.

In this section an evaluation has been carried out, based on data provided by highly reliable and precise sources. Table 1 shows two fundamental parameters for the evaluation of solar potential (Horizontal solar irradiation and direct normal irradiation), and, in addition to this, the potential related to the production of voltage from the use of photovoltaic panels. This is because the use of this type of renewable source is mostly linked to the production of electricity for use in lighting environments, considered important in the context of museums. In the above-mentioned table, it is evident that the intensities of the sites under study are low, but are within the limits allowed for their use in photovoltaic and thermal systems (heating), because they are geographically located on the coast of Peru, between 0 and 45 m above sea level, considering that altitude is an important factor for the intensity of solar radiation to be higher. It is also possible to note that the geographical area corresponding to the museums of "Royal Tombs of Sipan” and “Túcume" have the highest values of direct normal solar radiation, because their latitude is closer to the Earth's equator (northern area of Peru).

**Table 1:** Table of horizontal solar and normal direct irradiation, and photovoltaic production, based on the location of the museums under study.

Museum	Horizontal solar irradiation (KWh/m^2^) x day	Normal direct irradiation (KWh/m^2^) x day	Photovoltaic production (KWh/KWp) x day
On-site Museum Huacas de Moche	5.287	3.713	4.293
Cao Museum	5.563	3.94	4.567
Royal Tombs of Sipan Museum	5.736	4.441	4.628
Túcume Museum	5.633	4.351	4.478

Source: Own elaboration based on data taken from SOLARGIS.

**Figure 1:**
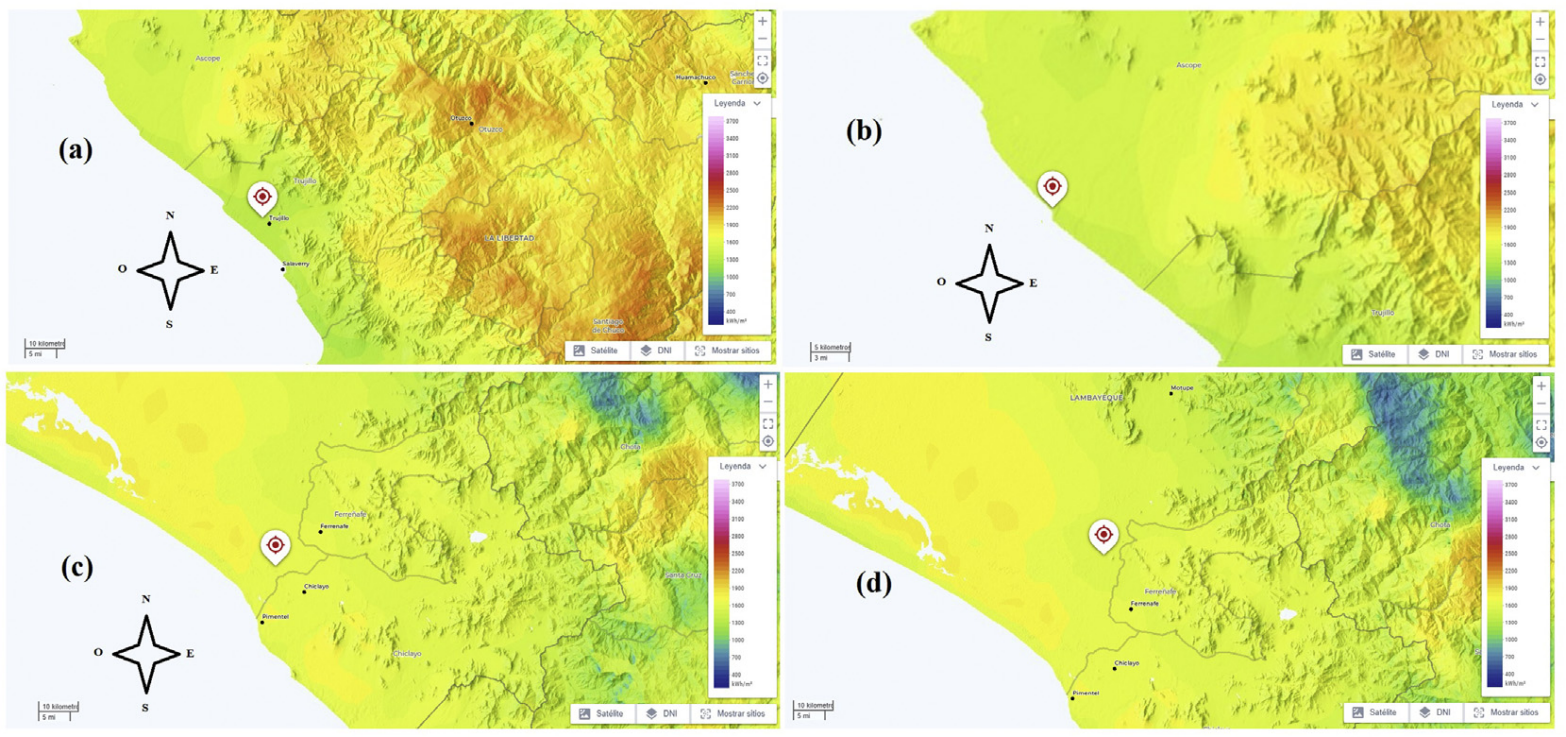
Normal direct irradiation of geographical sectors related to the museums under study. (A) On-site Museum Huacas de Moche, (B) Cao Museum, (C) Royal Tombs of Sipan Museum, (C) Túcume Museum. Source: SOLARGIS.

In Figure 1, satellite images of sectoral geographical values of normal direct irradiation are shown, indicating the points where the museums under study are located. Note the gradual increase in intense yellow coloration indicating small increases in the value of solar radiation intensity, and, in turn, the location sector near the coast of the Pacific Ocean.

#### 7.1.2 Wind potential

Another vital renewable resource that is being promoted is wind energy, whose energy efficiency is considered one of the most powerful and cost-effective ways to meet the demands of sustainable development (Velazquez, 2008). It is considered a great alternative for the generation of electricity from the social and environmental point of view (Xie and Billinton, 2011). In this context, diverse geographical factors influencing or determining wind intensity and frequency should be considered (James F. Manwell et al., 2010).

As mentioned, the applications are generally linked to the generation of electricity. However, currently there are architectural designs that allow to consolidate ventilation systems by turbo extractor and the management of humidity levels, factors considered important in the infrastructure of museums. In this way ideal temperatures for the preservation of various archaeological pieces with a value of 21 ± 2 °C (Rogers et al., 2013), and, in relation to the relative humidity of the environments (RH), with a value of 45 ± 8% (Padfield et al., 2007) are usually considered.

**Figure 2:**
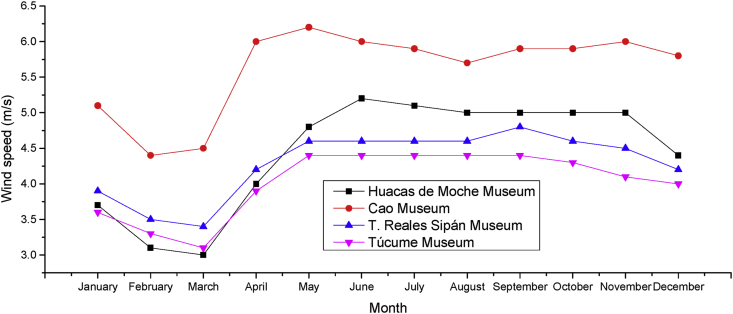
Annual wind speed variation based on geographic location of sites under study. Source: Own elaboration based on data acquired from EnAir.

Figure 2 summarizes the variation of wind speed according to the months of the year in each geographical sector under study. The results in this context are more encouraging, because the contribution of energy potential is much greater than the case of solar energy. This is possible because the location of each geographic site under study is close to the Pacific Ocean (Pacific coast of Peru). Among all the sites, the geographical context corresponding to the "Cao Museum" has high levels of contribution of wind potential.

With this type of energy, it would be possible to develop electricity generation systems through wind generators, as well as to provide a ventilation and moisture management system in museums.

### 7.2 Assessment of individual renewable potential based on the site under study

#### 7.2.1 On-site Museum Huacas de Moche

Among the 4 most outstanding museums in the north of Peru, and whose study is part of this manuscript, the one which has a modern architecture and a minimum percentage of use of renewable energy sources is the on-site museum "Huacas de Moche", located in the province of Trujillo, in the department of La Libertad in Peru. The use of renewable sources is linked to the fact that it has an air renewal system by turbo extractor, a device that works with wind energy. Likewise, the lighting of common environments is mostly gotten by indirect sunlight, which allows to reduce electricity consumption. However, there are elements that still depend on conventional electricity supply which is still higher. For this, in Table 2 the measurement of solar potential by using two measuring sources has been considered, obtaining an average of 4,003 KWh/day, which is considered low, but sufficient to think about the use of photovoltaic panels as a suggestion, and, to a lesser extent, a solar heating system for the environments of this site.

**Table 2:** Normal solar radiation intensity based on various global monitoring databases, corresponding to the geographical sector of the on-site museum Huacas de Moche.

Data	Solar radiation intensity (KWh/day)
EnAir	4.293
SOLARGIS	3.713
**AVERAGE**	**4.003**

**Source**: Own elaboration based on data collected and simulations of EnAir and SOLARGIS.

With respect to wind potential, the context is different, since in Table 2 it is possible to determine the noticeable difference of energy contribution of solar versus wind potentials, whose value is 29,486 KWh/year, sufficient amount to motivate the use of wind turbine power generation systems, and, thereby, decrease the use of other conventional energy sources. Table 3 presents a comparative graph of solar-wind energy contributions by months.

**Table 3:** Annual solar and wind production (power) of the geographical sector corresponding to the on-site museum Huacas de Moche.

ANUAL AVERAGE	
Type of energy	Value (KWh/year)
Solar production	1,285
Wind production	29,486

**Source:** EnAir.

#### 7.2.2 Cao Museum

This complex is located in the department of La Libertad, province of Ascope, north of the aforementioned museum, a place of modern infrastructure that exhibits a series of relics of high value. Currently, it does not have sustainable architecture. However, the design partially links the use of indirect solar energy with the aim of lighting some environments, but there are situations where solar radiation directly affects some relics, which can suffer irreparable damage over time.

Table 4 considers two sources of measurement for the assessment of solar potential, resulting in an average value of 4,567 KWh/day, the minimum potential needed to, in some months of the year (summer), take advantage of its use with photovoltaic panels.

**Table 4:** Normal solar radiation intensity based on various global monitoring databases, corresponding to the geographical sector of the Cao museum.

Data	Solar radiation intensity (KWh/day)
EnAir	4.567
SOLARGIS	4.567
**AVERAGE**	**4.567**

**Source**: Own elaboration based on data collected and simulations of EnAir and SOLARGIS.

The wind potential in this geographic site makes this one of the places where this renewable source is quite high, reaching 46554 KWh/year, a value considered the highest compared to the other geographic sectors under study, an ideal place for its best use in the generation of electricity by wind generators, and indirect ventilation in the museum. Table 5 shows a comparison of solar-wind energy production corresponding to the geographical location according to the months of the year.

**Table 5:** Annual solar and wind production (power) of the geographical sector corresponding to the Cao museum.

ANUAL AVERAGE	
Type of energy	Value (KWh/year)
Solar production	1,367
Wind production	46,554

**Source:** EnAir.

#### 7.2.3 Royal Tombs of Sipan Museum

This museum is located in the department of Lambayeque, in the northern part of Peru, a geographical place closer to the Earth's equator, which gives it a slightly higher intensity of solar radiation than that of the museums located in La Libertad region. The analysis results in an average of 4.5345 KWh/day of intensity of solar radiation, which allows better efficiency in the use of photovoltaic panels, solar thermal heating systems, and, therefore, better contribution in the energy demand of the site (Table 6).

**Table 6:** Normal solar radiation intensity based on various global monitoring databases, corresponding to the geographical sector of the Royal Tombs of Sipan museum.

Data	Solar radiation intensity (KWh/day)
EnAir	4.628
SOLARGIS	4.441
**AVERAGE**	**4.5345**

**Source**: Own elaboration based on data collected and simulations of EnAir and SOLARGIS.

Regarding wind potential, it also has an excellent contribution, which is well above solar potential and whose value is 24,996 KWh/year. In Table 7 a comparison between both types of renewable energies is indicated.

**Table 7:** Annual solar and wind production (energy) of the geographical sector corresponding to the Royal Tombs of Sipan Museum.

ANUAL AVERAGE	
Type of energy	Value (KWh/year)
Solar production	1,387
Wind production	24,996

**Source:** EnAir.

#### 7.2.4 Túcume Museum

The geographical space of this museum is similar to that of the Royal Tombs of Sipan Museum, because both museums are in the same geographical region; therefore, the values of energy potential are similar. The average values per day of solar radiation intensity is 4.4145 KWh/day (Table 8), sufficient for its use in photovoltaic systems.

**Table 8:** Normal solar radiation intensity based on various global monitoring databases, corresponding to the geographical sector of the Túcume museum.

Data	Solar radiation intensity (KWh/day)
EnAir	4.478
SOLARGIS	4.351
**AVERAGE**	**4.4145**

**Source**: Own elaboration based on data collected and simulations of EnAir and SOLARGIS.

As far as wind potential is concerned, the average annual value is the lowest among the other three sites under study, but, still, it is a sufficient amount, since it would contribute to the energy level with 22,157 KWh/year. (Table 9)

**Table 9:** Annual solar and wind production (energy) of the geographical sector corresponding to the Túcume Museum.

ANUAL AVERAGE	
Type of energy	Value (KWh/year)
Solar production	1,339
Wind production	22,157

**Source:** EnAir.

**Figure 3:**
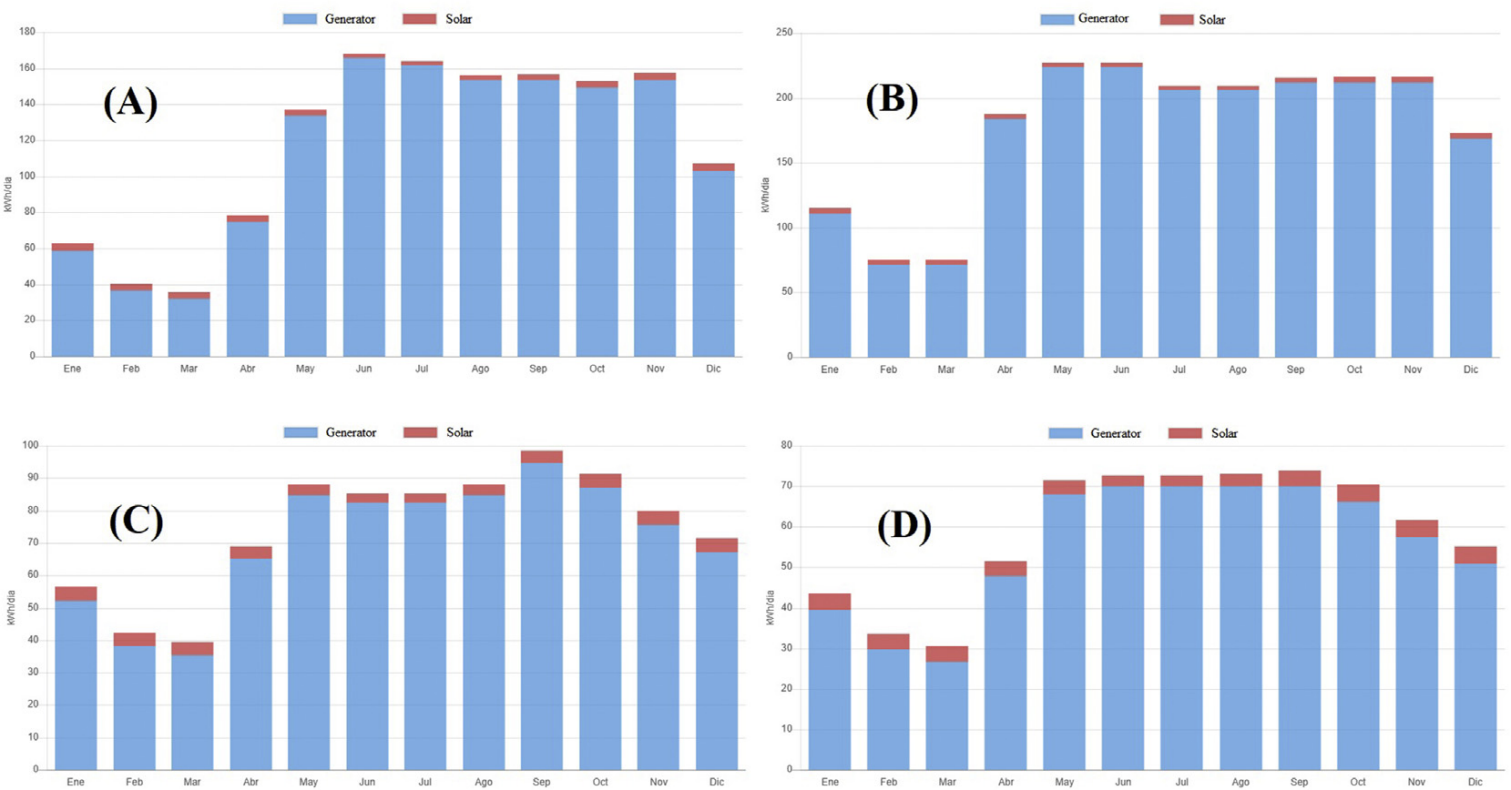
Estimated annual wind (blue) and solar (red) production (energy) of the geographical sector corresponding to: (A) On-site Museum Huacas de Moche, (B) Cao Museum, (C) Royal Tombs of Sipan Museum, (D) Túcume Museum.

Figure 3 summarizes the sum of both solar and wind potentials according to the site under study. It can be observed that wind potential (blue) is that one with the greatest energy contribution in every sectors, which implies a total of 123,193 KWh/year, or a monthly average of 10,266.08 KWh/month, which is equivalent to a CO_2_ emission savings of 32.95 ton/year (Table 10). By contrast, solar potential (red), as the sum of the 4 sites under study, is 5378 KWh/year, which is equivalent to a CO_2_ emission savings of 1.45 ton/year. This data would be a consequence of the use of the existing renewable energy potential in the museums under study, promoting a reduction in costs of conventional supply energy consumption, contributing with the context of sustainability, reducing greenhouse gas emissions, among others.

**Table 10:** Equivalence of CO_2_ savings (ton/year), as a consequence of the renewable energy potential (solar and wind) in the museums under study.

Museum	Type of CO_2_ savings	Value (Ton/year)
On-site Museum Huacas de Moche	Solar	0.35
	Wind	7.96
Cao Museum	Solar	0.37
	Wind	12.57
Royal Tombs of Sipan Museum	Solar	0.37
	Wind	6.44
Túcume Museum	Solar	0.36
	Wind	5.98

Source: Own elaboration based on data acquired from EnAir.

As is known, climate change threatens the future of the planet; in recent years, various natural phenomena have occurred that are affecting millions of people, in addition to causing numerous material damage and the displacement of people (IDMC, 2020), this as a result of a long process of environmental contamination, in that sense, it is our responsibility, and that of the government sector, to implement in all its development instances, policies that have a strong link with the controlled use of its renewable energy sources, this in order to mitigate future effects. At a global level, efforts have been focused on trying to limit greenhouse gas emissions, but the latest data attest to a worrying impact, this is how the international community has been diversifying its efforts with the aim of promoting policies that are not so only mitigation, but also adaptation to climate change. Among the mitigation measures are to improve energy efficiency and bet on renewable energies, in addition to promoting the construction of more sustainable infrastructures (sustainable architecture). This research consolidates and promotes the strengthening of public and governmental policies, so that they are used in various activities that the country promotes, being tourism, and specifically its application in the enabling of sustainable museums, a part of it, because according to Table 10, annually this would be translated into a reduction of 34.4 tons/year of CO_2_, with only four museums, out of a total of 56 in Peru, becoming a contribution to mitigating climate change.

## 8 Conclusions

The statistics show a constant growth in tourist activity; the northern part of Peru where the most important archaeological museums are located is not alien to this growth. In this sense, the results of the review of the literature of the Peruvian legal framework reveal a lack of laws and regulations specifically focused on sustainable tourism, however, there is a motivation to develop a more sustainable country, since various government entities and not government have launched initiatives that aim to direct plans related to laws that contribute to achieving tourism sustainability, for the moment it has resulted in specific regulations that are linked only to promoting sustainable tourism. Regarding the renewable sources of the country in question, the existing potential is known, however, the legal regulations are not specific and much less generate a link with tourist activity, the legislation in this aspect is very general and focuses on promoting the implementation of renewable energy elements for rural electrification, and not direct it to tourism, which as has been shown is an important economic sector in the country. The quantified evaluation of the solar and wind potentials of the geographical area under study indicates that renewable energy potential available for transformation and use in the development of sustainable museums and thus reduce environmental impact, contribute to the reduction of the carbon footprint. and sustainable tourism development. It is worth mentioning that, according to data from MINCETUR, the new profile of the national and foreign visitor/tourist has great expectations in visiting centers that have a sustainable profile; an improvement in the legal framework under discussion and putting into practice the use of its energy resources, would imply an innovation in its tourism offer.

## Declarations

### Author contribution statement

Calderón-Vargass, F.: Conceived and designed the experiments; Performed the experiments; Wrote the paper.

Asmat-Campos, D.: Conceived and designed the experiments; Performed the experiments; Analyzed and interpreted the data; Wrote the paper.

Chávez-Arroyo, P.: Contributed reagents, materials, analysis tools or data; Wrote the paper.

### Funding statement

This research did not receive any specific grant from funding agencies in the public, commercial, or not-for-profit sectors.

### Data availability statement

Data included in article.

### Declaration of interests statement

The authors declare no conflict of interest.

### Additional information

No additional information is available for this paper.
